# (1-Adamant­yl)(2-methyl­phen­yl)methanone

**DOI:** 10.1107/S160053681004818X

**Published:** 2010-11-24

**Authors:** Eva Babjaková, Marek Nečas, Robert Vícha

**Affiliations:** a Department of Chemistry, Faculty of Technology, Tomas Bata University in Zlin, Nám. T. G. Masaryka 275, Zlín,762 72, Czech Republic; b Department of Chemistry, Faculty of Science, Masaryk University in Brno, Kamenice 5, Brno-Bohunice, 625 00, Czech Republic

## Abstract

In the title compound, C_18_H_22_O, the dihedral angle between the carbonyl and benzene planes is 69.11 (6)°. In the adamantyl group, the three fused cyclo­hexane rings have almost ideal chair conformations, with C—C—C angles in the range 108.14 (11)–110.50 (11)°. No specific inter­molecular inter­actions (other than van der Waals inter­actions) are present in the crystal.

## Related literature

For background to the synthesis, see: Vícha *et al.* (2006 ▶); Austin & Johnson (1932 ▶). For an alternative method for the preparation of the title compound, see: Lo Fiego *et al.* (2009 ▶).
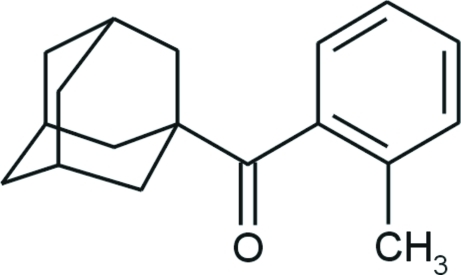


## Experimental

### 

#### Crystal data

C_18_H_22_O
*M* *_r_* = 254.36
Monoclinic, 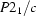

*a* = 6.6988 (4) Å
*b* = 12.2971 (6) Å
*c* = 16.7670 (7) Å
β = 92.244 (4)°
*V* = 1380.14 (12) Å^3^
*Z* = 4
Mo *K*α radiation
μ = 0.07 mm^−1^
*T* = 120 K
0.40 × 0.40 × 0.30 mm

#### Data collection

Oxford Diffraction Xcalibur diffractometer with a Sapphire2 detector
Absorption correction: multi-scan (*CrysAlis RED*; Oxford Diffraction, 2009 ▶) *T*_min_ = 0.974, *T*_max_ = 1.000
8111 measured reflections
2414 independent reflections
1673 reflections with *I* > 2σ(*I*)
*R*_int_ = 0.029

#### Refinement

*R*[*F* ^2^ > 2σ(*F* ^2^)] = 0.037
*wR*(*F* ^2^) = 0.084
*S* = 0.96
2414 reflections
173 parameters
H-atom parameters constrained
Δρ_max_ = 0.17 e Å^−3^
Δρ_min_ = −0.19 e Å^−3^

### 

Data collection: *CrysAlis CCD* (Oxford Diffraction, 2009 ▶); cell refinement: *CrysAlis RED* (Oxford Diffraction, 2009 ▶); data reduction: *CrysAlis RED*; program(s) used to solve structure: *SHELXS97* (Sheldrick, 2008) ▶; program(s) used to refine structure: *SHELXL97* (Sheldrick, 2008) ▶; molecular graphics: *ORTEP-3* (Farrugia, 1997 ▶) and *Mercury* (Macrae *et al.*, 2008 ▶); software used to prepare material for publication: *SHELXL97* ▶.

## Supplementary Material

Crystal structure: contains datablocks global, I. DOI: 10.1107/S160053681004818X/pk2284sup1.cif

Structure factors: contains datablocks I. DOI: 10.1107/S160053681004818X/pk2284Isup2.hkl

Additional supplementary materials:  crystallographic information; 3D view; checkCIF report

## Figures and Tables

**Fig. 1.**
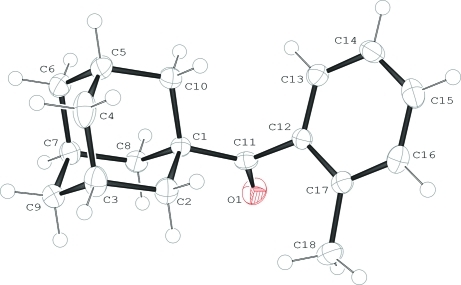
An ellipsoid plot of the asymmetric unit with atoms represented as 50% probability ellipsoids.

**Fig. 2.**
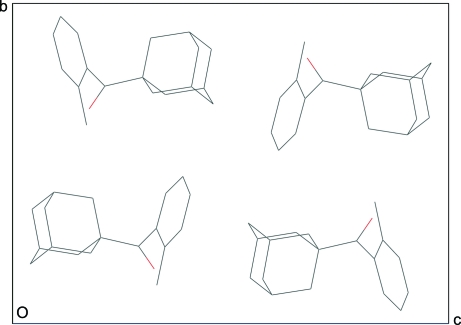
Part of the crystal structure showing unit cell projected along the a-axis. H-atoms have been omitted for clarity.

## supplementary crystallographic information

### Comment

The title compound arose from the reaction of adamantane-1-carbonyl chloride with benzylmagnesium chloride as a product of the rearrangement of a starting Grignard reagent. Similar behavior of benzylmagnesium halides has been described previously (Austin & Johnson, 1932). Alternatively, the title compound may be prepared by the reaction of adamantane-1-carbonyl chloride with 2-methylphenyl(tributyl)stannane as Lo Fiego *et al.* (2009) have described. In the molecule of the title compound (Fig. 1), the angle between carbonyl plane P1 (C1, C11, C12, O1) and benzene ring plane P2 (C12–C17) is 69.11 (6)°. Such a large twist may be attributed to the steric hindrance between the bulky adamantane moiety and the benzene ring. Nevertheless, the carbon of the methyl group in the *ortho* position is located almost in the ring plane with a deviation of 0.0587 (15) Å. Maximum deviations from the best planes are 0.0229 (13)Å for C11 and -0.0132 (13)Å for C12, respectively. No specific intermolecular interactions were observed in crystal packing.

### Experimental

The title compound was prepared by the reaction of adamantane-1-carbonyl chloride with benzylmagnesium chloride according to the procedure published previously (Vícha *et al.*, 2006). The colorless microcrystalline powder was isolated from a crude complex mixture by column chromatography (silicagel; petroleum ether/ethyl acetate, *v*/*v*, 16/1). A single-crystal for X-ray analysis was acquired by spontaneous evaporation from deuterochloroform at room temperature.

### Refinement

H atoms were found in difference Fourier maps and subsequently placed in idealized positions with constrained distances of 0.98 Å (RCH_3_), 0.99 Å (R_2_CH_2_), 1.00 Å (R_3_CH), 0.95 Å (C_Ar_H), and with U_iso_(H) values set to either 1.2U_eq_ or 1.5U_eq_ (RCH_3_) of the attached atom.

### Crystal data

**Table:**

C_18_H_22_O	*F*(000) = 552
*M**_r_* = 254.36	*D*_x_ = 1.224 Mg m^−^^3^
Monoclinic, *P*2_1_/*c*	Melting point: 345 K
Hall symbol: -P 2ybc	Mo *K*α radiation, λ = 0.71073 Å
*a* = 6.6988 (4) Å	Cell parameters from 2705 reflections
*b* = 12.2971 (6) Å	θ = 3.3–27.3°
*c* = 16.7670 (7) Å	µ = 0.07 mm^−^^1^
β = 92.244 (4)°	*T* = 120 K
*V* = 1380.14 (12) Å^3^	Block, colourless
*Z* = 4	0.40 × 0.40 × 0.30 mm

### Data collection

**Table:**

Oxford Diffraction Xcalibur diffractometer with a Sapphire2 detector	2414 independent reflections
Radiation source: Enhance (Mo) X-ray Source	1673 reflections with *I* > 2σ(*I*)
graphite	*R*_int_ = 0.029
Detector resolution: 8.4353 pixels mm^-1^	θ_max_ = 25.0°, θ_min_ = 3.5°
ω scan	*h* = −7→6
Absorption correction: multi-scan (*CrysAlis RED*; Oxford Diffraction, 2009)	*k* = −14→14
*T*_min_ = 0.974, *T*_max_ = 1.000	*l* = −19→19
8111 measured reflections	

### Refinement

**Table:**

Refinement on *F*^2^	Primary atom site location: structure-invariant direct methods
Least-squares matrix: full	Secondary atom site location: difference Fourier map
*R*[*F*^2^ > 2σ(*F*^2^)] = 0.037	Hydrogen site location: inferred from neighbouring sites
*wR*(*F*^2^) = 0.084	H-atom parameters constrained
*S* = 0.96	*w* = 1/[σ^2^(*F*_o_^2^) + (0.0418*P*)^2^] where *P* = (*F*_o_^2^ + 2*F*_c_^2^)/3
2414 reflections	(Δ/σ)_max_ < 0.001
173 parameters	Δρ_max_ = 0.17 e Å^−^^3^
0 restraints	Δρ_min_ = −0.19 e Å^−^^3^

### Special details

**Table:**

Geometry. All e.s.d.'s (except the e.s.d. in the dihedral angle between two l.s. planes) are estimated using the full covariance matrix. The cell e.s.d.'s are taken into account individually in the estimation of e.s.d.'s in distances, angles and torsion angles; correlations between e.s.d.'s in cell parameters are only used when they are defined by crystal symmetry. An approximate (isotropic) treatment of cell e.s.d.'s is used for estimating e.s.d.'s involving l.s. planes.
Refinement. Refinement of *F*^2^ against ALL reflections. The weighted *R*-factor *wR* and goodness of fit *S* are based on *F*^2^, conventional *R*-factors *R* are based on *F*, with *F* set to zero for negative *F*^2^. The threshold expression of *F*^2^ > σ(*F*^2^) is used only for calculating *R*-factors(gt) *etc*. and is not relevant to the choice of reflections for refinement. *R*-factors based on *F*^2^ are statistically about twice as large as those based on *F*, and *R*- factors based on ALL data will be even larger.

### Fractional atomic coordinates and isotropic or equivalent isotropic displacement parameters (Å^2^)

**Table:**

	*x*	*y*	*z*	*U*_iso_*/*U*_eq_	
O1	0.03229 (15)	0.67052 (8)	0.17539 (6)	0.0327 (3)	
C1	0.0839 (2)	0.76864 (11)	0.29798 (8)	0.0198 (3)	
C2	0.2536 (2)	0.71524 (12)	0.34999 (8)	0.0275 (4)	
H2A	0.2572	0.6361	0.3393	0.033*	
H2B	0.3839	0.7466	0.3361	0.033*	
C3	0.2185 (2)	0.73491 (13)	0.43858 (9)	0.0311 (4)	
H3	0.3283	0.7000	0.4716	0.037*	
C4	0.2157 (2)	0.85708 (14)	0.45562 (9)	0.0362 (4)	
H4A	0.1940	0.8697	0.5130	0.043*	
H4B	0.3458	0.8896	0.4428	0.043*	
C5	0.0483 (2)	0.91065 (12)	0.40506 (9)	0.0309 (4)	
H5	0.0471	0.9906	0.4161	0.037*	
C6	−0.1514 (2)	0.86164 (12)	0.42657 (9)	0.0292 (4)	
H6A	−0.2608	0.8966	0.3945	0.035*	
H6B	−0.1747	0.8748	0.4837	0.035*	
C7	−0.1501 (2)	0.73938 (12)	0.41004 (8)	0.0247 (4)	
H7	−0.2811	0.7073	0.4243	0.030*	
C8	−0.1164 (2)	0.71996 (12)	0.32129 (8)	0.0244 (4)	
H8A	−0.2263	0.7538	0.2888	0.029*	
H8B	−0.1173	0.6409	0.3102	0.029*	
C9	0.0183 (2)	0.68552 (12)	0.46001 (9)	0.0293 (4)	
H9A	−0.0036	0.6968	0.5175	0.035*	
H9B	0.0192	0.6063	0.4495	0.035*	
C10	0.0831 (2)	0.89173 (11)	0.31629 (8)	0.0268 (4)	
H10A	0.2126	0.9240	0.3024	0.032*	
H10B	−0.0239	0.9277	0.2835	0.032*	
C11	0.1261 (2)	0.74157 (11)	0.21138 (8)	0.0217 (3)	
C12	0.2950 (2)	0.79751 (11)	0.17062 (8)	0.0200 (3)	
C13	0.2765 (2)	0.90683 (12)	0.14885 (8)	0.0262 (4)	
H13	0.1590	0.9456	0.1611	0.031*	
C14	0.4271 (2)	0.95941 (12)	0.10975 (9)	0.0306 (4)	
H14	0.4125	1.0336	0.0947	0.037*	
C15	0.5985 (2)	0.90312 (13)	0.09284 (8)	0.0312 (4)	
H15	0.7042	0.9390	0.0673	0.037*	
C16	0.6163 (2)	0.79443 (12)	0.11302 (8)	0.0269 (4)	
H16	0.7346	0.7565	0.1006	0.032*	
C17	0.4658 (2)	0.73909 (11)	0.15109 (8)	0.0218 (3)	
C18	0.4921 (2)	0.61964 (12)	0.16948 (9)	0.0297 (4)	
H18A	0.6308	0.5985	0.1613	0.045*	
H18B	0.4026	0.5770	0.1340	0.045*	
H18C	0.4598	0.6059	0.2251	0.045*	

### Atomic displacement parameters (Å^2^)

**Table:**

	*U*^11^	*U*^22^	*U*^33^	*U*^12^	*U*^13^	*U*^23^
O1	0.0341 (7)	0.0331 (6)	0.0309 (6)	−0.0089 (5)	0.0016 (5)	−0.0102 (5)
C1	0.0196 (8)	0.0194 (8)	0.0203 (8)	0.0001 (6)	0.0004 (6)	0.0000 (6)
C2	0.0223 (9)	0.0350 (9)	0.0253 (9)	0.0044 (7)	0.0000 (7)	0.0016 (7)
C3	0.0246 (9)	0.0471 (11)	0.0213 (8)	0.0065 (8)	−0.0027 (7)	0.0032 (7)
C4	0.0332 (10)	0.0530 (12)	0.0227 (9)	−0.0160 (8)	0.0029 (8)	−0.0080 (8)
C5	0.0445 (11)	0.0230 (9)	0.0258 (9)	−0.0058 (7)	0.0082 (8)	−0.0059 (7)
C6	0.0322 (10)	0.0299 (9)	0.0259 (9)	0.0060 (7)	0.0047 (7)	0.0001 (7)
C7	0.0212 (9)	0.0276 (9)	0.0255 (8)	−0.0032 (7)	0.0031 (7)	0.0017 (7)
C8	0.0231 (9)	0.0233 (8)	0.0266 (8)	−0.0033 (6)	−0.0006 (7)	−0.0009 (6)
C9	0.0359 (10)	0.0277 (9)	0.0247 (8)	0.0044 (7)	0.0050 (7)	0.0033 (7)
C10	0.0339 (10)	0.0210 (8)	0.0258 (8)	−0.0035 (7)	0.0039 (7)	−0.0006 (6)
C11	0.0215 (8)	0.0185 (8)	0.0248 (8)	0.0040 (7)	−0.0041 (7)	0.0007 (6)
C12	0.0243 (9)	0.0206 (8)	0.0150 (7)	−0.0006 (6)	−0.0020 (6)	−0.0010 (6)
C13	0.0317 (9)	0.0245 (9)	0.0224 (8)	0.0036 (7)	0.0019 (7)	−0.0006 (6)
C14	0.0459 (11)	0.0214 (9)	0.0245 (9)	−0.0030 (7)	0.0026 (8)	0.0029 (6)
C15	0.0341 (10)	0.0357 (10)	0.0240 (8)	−0.0093 (8)	0.0040 (7)	0.0002 (7)
C16	0.0229 (9)	0.0356 (10)	0.0221 (8)	0.0013 (7)	0.0013 (7)	−0.0034 (7)
C17	0.0246 (9)	0.0239 (8)	0.0167 (7)	0.0012 (6)	−0.0028 (6)	−0.0020 (6)
C18	0.0313 (9)	0.0263 (9)	0.0315 (9)	0.0065 (7)	−0.0001 (7)	−0.0024 (7)

### Geometric parameters (Å, °)

**Table:**

O1—C11	1.2219 (16)	C7—H7	1.0000
C1—C11	1.5268 (18)	C8—H8A	0.9900
C1—C8	1.5338 (19)	C8—H8B	0.9900
C1—C10	1.5445 (18)	C9—H9A	0.9900
C1—C2	1.551 (2)	C9—H9B	0.9900
C2—C3	1.5321 (19)	C10—H10A	0.9900
C2—H2A	0.9900	C10—H10B	0.9900
C2—H2B	0.9900	C11—C12	1.5101 (19)
C3—C9	1.528 (2)	C12—C13	1.3972 (18)
C3—C4	1.530 (2)	C12—C17	1.4009 (19)
C3—H3	1.0000	C13—C14	1.3843 (19)
C4—C5	1.529 (2)	C13—H13	0.9500
C4—H4A	0.9900	C14—C15	1.380 (2)
C4—H4B	0.9900	C14—H14	0.9500
C5—C6	1.5232 (19)	C15—C16	1.383 (2)
C5—C10	1.5332 (19)	C15—H15	0.9500
C5—H5	1.0000	C16—C17	1.3918 (19)
C6—C7	1.529 (2)	C16—H16	0.9500
C6—H6A	0.9900	C17—C18	1.5097 (19)
C6—H6B	0.9900	C18—H18A	0.9800
C7—C9	1.529 (2)	C18—H18B	0.9800
C7—C8	1.5325 (18)	C18—H18C	0.9800
			
C11—C1—C8	110.71 (12)	C1—C8—H8A	109.6
C11—C1—C10	113.89 (11)	C7—C8—H8B	109.6
C8—C1—C10	108.78 (11)	C1—C8—H8B	109.6
C11—C1—C2	106.46 (11)	H8A—C8—H8B	108.1
C8—C1—C2	108.70 (11)	C3—C9—C7	109.53 (12)
C10—C1—C2	108.14 (12)	C3—C9—H9A	109.8
C3—C2—C1	109.96 (12)	C7—C9—H9A	109.8
C3—C2—H2A	109.7	C3—C9—H9B	109.8
C1—C2—H2A	109.7	C7—C9—H9B	109.8
C3—C2—H2B	109.7	H9A—C9—H9B	108.2
C1—C2—H2B	109.7	C5—C10—C1	110.09 (11)
H2A—C2—H2B	108.2	C5—C10—H10A	109.6
C9—C3—C4	109.22 (12)	C1—C10—H10A	109.6
C9—C3—C2	109.56 (13)	C5—C10—H10B	109.6
C4—C3—C2	109.85 (12)	C1—C10—H10B	109.6
C9—C3—H3	109.4	H10A—C10—H10B	108.2
C4—C3—H3	109.4	O1—C11—C12	118.83 (13)
C2—C3—H3	109.4	O1—C11—C1	120.98 (13)
C5—C4—C3	109.49 (12)	C12—C11—C1	120.07 (12)
C5—C4—H4A	109.8	C13—C12—C17	119.81 (13)
C3—C4—H4A	109.8	C13—C12—C11	119.76 (12)
C5—C4—H4B	109.8	C17—C12—C11	120.36 (12)
C3—C4—H4B	109.8	C14—C13—C12	120.96 (14)
H4A—C4—H4B	108.2	C14—C13—H13	119.5
C6—C5—C4	109.28 (12)	C12—C13—H13	119.5
C6—C5—C10	109.77 (13)	C15—C14—C13	119.38 (14)
C4—C5—C10	109.68 (13)	C15—C14—H14	120.3
C6—C5—H5	109.4	C13—C14—H14	120.3
C4—C5—H5	109.4	C14—C15—C16	119.94 (14)
C10—C5—H5	109.4	C14—C15—H15	120.0
C5—C6—C7	109.56 (12)	C16—C15—H15	120.0
C5—C6—H6A	109.8	C15—C16—C17	121.86 (14)
C7—C6—H6A	109.8	C15—C16—H16	119.1
C5—C6—H6B	109.8	C17—C16—H16	119.1
C7—C6—H6B	109.8	C16—C17—C12	117.99 (13)
H6A—C6—H6B	108.2	C16—C17—C18	119.27 (13)
C6—C7—C9	109.62 (12)	C12—C17—C18	122.74 (12)
C6—C7—C8	109.35 (11)	C17—C18—H18A	109.5
C9—C7—C8	109.33 (12)	C17—C18—H18B	109.5
C6—C7—H7	109.5	H18A—C18—H18B	109.5
C9—C7—H7	109.5	C17—C18—H18C	109.5
C8—C7—H7	109.5	H18A—C18—H18C	109.5
C7—C8—C1	110.50 (12)	H18B—C18—H18C	109.5
C7—C8—H8A	109.6		
